# Clinical Significance of Discordance between Hip and Spine Bone Mineral Density in Korean Elderly Patients with Hip Fractures

**DOI:** 10.3390/jcm12206448

**Published:** 2023-10-10

**Authors:** Se-Won Lee, Younghyun Yoon, Junhyuk Kwon, Jun-Young Heu, Jihyo Hwang

**Affiliations:** 1 Department of Orthopedic Surgery, Yeouido St. Mary’s Hospital, College of Medicine, The Catholic University of Korea, Seoul 07345, Republic of Korea; ssewon@naver.com; 2 Department of Orthopedic Surgery, Gangnam Sacred Heart Hospital, College of Medicine, Hallym University, Seoul 07441, Republic of Korea; mgyhh@naver.com (Y.Y.); junhyuck367@gmail.com (J.K.); 3 Department of Orthopedic Surgery, Incheon St. Mary’s Hospital, College of Medicine, The Catholic University of Korea, Incheon 21431, Republic of Korea; med@live.co.kr

**Keywords:** osteoporosis, hip fracture, bone mineral density, discordance

## Abstract

The clinical significance of BMD discordance has not yet been elucidated. The objective of this study was to evaluate the clinical significance of BMD discordance between the hip and spine for hip fractures. The BMD was measured and related factors were investigated in 109 elderly patients hospitalized for a hip fracture (fracture group) and 109 patients hospitalized without a hip fracture (non-fracture group). BMD discordance of the hip and spine was classified as minor discordance (normal and osteopenia, and osteopenia and osteoporosis) and major discordance (normal and osteoporosis). The risk of hip fracture was calculated according to the type of discordance: no discordance, low hip, and lower spine. There was no significant difference between the general characteristics of the fracture group and the non-fracture group. The rate of BMD discordance and low hip discordance were significantly higher in the fracture group (53.2%, 43.1%) than in the non-fracture group (28.4%, 19.3%). The odds ratio of hip fracture was 2.86 times higher in patients with BMD discordance than in those without discordance and 3.42 times higher in the patients with low hip discordance than in those without no hip discordance. The presence of discordance, particularly when there is low hip discordance, might be related to the hip fractures.

## 1. Introduction

In South Korea, the prevalence of osteopenia in adults over 50 years of age is 47.9% and the prevalence of osteoporosis is 22.4% [1]. In particular, the prevalence of osteoporosis in women over 70 years of age is 68.5% [1]. The number of osteoporotic fractures in adults over 50 years of age were 186,488 cases in 2008, 274,414 cases in 2013, and 275,131 cases in 2018. The number of osteoporotic fractures did not decrease despite the prescription of many types of osteoporosis drugs [1,2,3]. Early prevention is important as a decrease in bone density can lead to a second fracture [4]. A vertebral fracture is the most common among osteoporotic fractures in the elderly, occurring even with low-energy trauma; thus, the fracture may not be recognized. In many cases, treatment is not performed because of a deficit in proper diagnoses, and it can be an important indicator for predicting second fractures [5]. Conversely, the possibility of hip fracture occurrence increases rapidly after the age of 70 years due to falls, and as it is difficult to walk after an accident, this often leads to emergency room visits. Even if it is relatively easy to diagnose, the annual mortality rate of hip fracture is approximately 20–40%; therefore, it is known as a fatal fracture, unlike vertebral fractures [6].

The first step to prevent osteoporotic fractures is to diagnose osteoporosis through tests such as plain X-ray, computed tomography (CT), ultrasound scanning, and dual energy X-ray absorptiometry (DXA). Among them, DXA is the standard test for measuring bone mineral density (BMD) [7]. It can measure the bone density of a single site or multiple sites, with the most significant parts, the lumbar spine (spine) and hip, measured sequentially. Osteoporosis is diagnosed and treated on the basis of the T-score of the site with the lower BMD.

However, sometimes there is a difference in BMDs between the two, which may confuse interpretation and treatment. In other words, there are cases in which osteoporosis occurs only in the spine, while the hip bone density is normal. Conversely, in some cases, the vertebral BMD is normal, but the femoral BMD is osteoporotic. Therefore, the treatment should be more oriented to the spine or hip. A hip surgeon should emphasize the proper treatment in the patients who have a low hip discordance.

Therefore, the purpose of this study was to (1) analyze the epidemiology of T-score discordance between the spine and femur and (2) compare the prevalence of T-score discordance between patients with hip fractures and patients without hip fractures from a single institution using 1:1 propensity score matching. We hypothesized that the prevalence of discordance of T-score between lumbar spine (LS) and femur neck (FN) would be significantly higher in patients with hip fractures than in patients without hip fractures.

## 2. Materials and Methods

### 2.1. Study Population

This study was conducted on elderly patients aged 65 years or older who were admitted to Gangnam Sacred Heart Hospital. BMD was analyzed through DXA in 109 elderly patients who underwent hip fracture surgery and 109 patients hospitalized without hip fracture between 2015 and 2021.

Hip fractures occur around the hip joint, including femoral head fractures, femoral neck fractures, trochanteric fractures, and subtrochanteric fractures. Only patients with the femoral neck and intertrochanteric fractures, which are senile hip fractures, were included in this study and defined as the hip fracture group. Only patients whose BMD was measured at two sites in the DXA test were included. The DXA test was performed prior to surgery, or post-surgery. We excluded cases in which the period between the fracture and the measurement of BMD was more than 1 year. The control group was selected from the pool of patients from 450 consecutive patients who had been hospitalized between 2015 and 2021 for any reasons except hip fractures, including degenerative knee osteoarthritis, degenerative spine disease, ankle fractures, and so on. Confounding factors included sex, age, and body mass index (BMI). Based on these variables, 1:1 propensity score matching patient match was carried out using propensity score estimated on the scale of the log odds under a logistic regression mode. This patient group was defined as the non-fracture group.

### 2.2. Measurement of BMD

BMD was determined using the method suggested in the “Physician’s Guide for Osteoporosis 2020”, published by the Korean Society of Bone and Mineral Research. DXA is the most common test, which is the gold standard for measuring BMD, and the BMD measuring device used for this study was Horizon W (S/N200630, Hologic, Inc, Budford, MA, USA) [8]. Furthermore, the average value of lumbar vertebrae 1 to 4 was measured, and the average value of two or more sites was used, excluding sites that were frequently measured due to degenerative changes. In the case of the hip, the ROIs (regions of interest) were divided and measured as neck, trochanter, intertrochanter, and total, and the lowest T-score or BMD (g/cm^2^) was selected. In this study, we compared the difference between hip BMD and spine BMD, which are likely to have a significant impact on hip fracture. Osteoporosis was analyzed based on T-scores, with a T-score of −1.0 or higher defined as normal, −1.0 > T-score > −2.5 as osteopenia, and below −2.5 as osteoporosis [9].

The discordance in BMD was determined as normal versus osteopenia, normal versus osteoporosis, and osteopenia versus osteoporosis, in the lumbar and hip regions, respectively. The extent of discordance in the fracture and non-fracture groups was compared, and the classification of discordance was divided into no discordance, minor discordance, and major discordance. Major discordance was defined as normal at one site and osteoporosis at another site, and minor discordance was defined as normal at one site and osteopenia at another or osteopenia at one site and osteoporosis at another [10].

We also analyzed the type of discordance, and the cases were divided into no discordance, lower hip discordance (low hip discordance), and lower spine discordance (low spine discordance). Diabetic mellitus, hypertension, hemodialysis, rheumatoid arthritis, smoking, and drinking were identified as underlying diseases, and correlations were analyzed. If the patients drink more than one cup (1/7 bottle) of Soju every day, this means 57.2 gm/week of ethanol, which we defined as alcohol drinker.

### 2.3. Statistical Analysis

According to the normal distribution, Student’s *t*-test or Mann–Whitney U test was used to compare continuous data, and the χ test or Fisher’s exact test was used to analyze categorical data. For all other tests, a 2-sided *p*-value of 0.05 was considered significant. To determine the risk factors of discordance, multivariable regression analysis was performed on variables with a *p*-value less than 0.1. The odds ratio (OR) and 95% confidence interval (CI) were calculated to examine the risk of hip fracture according to the presence or absence of BMD discordance. The sample size was calculated using discordance rates from the existing literature in ‘clincal.com’ with an alpha value of 0.5, power of 95%, and enrollment ratio of 1. Consequently, the minimum sample size was calculated to be 45 for each group. Statistical analyses were conducted with the SPSS for Windows statistical package, version 16.0 (SPSS Inc., Chicago, IL, USA). The significance level was set at *p* < 0.05.

## 3. Results

### 3.1. Clinical Characteristics of the Study Population

The mean age of the fracture group was 77.9 years, while that of the non-fracture group was 76.9 years (*p* = 0.284). The fracture group included 36 males (33.0%) and 73 females (67.0%), and the non-fracture group comprised 38 men (34.9%) and 71 women (65.1%) (*p* = 0.775). The average height was 158.5 cm in the fracture group and 157.8 cm in the non-fracture group (*p* = 0.499), and the average weight was 55.9 kg in the fracture group and 57.2 kg in the non-fracture group (*p* = 0.334). The mean body mass index (BMI, kg/m^2^) was 22.3 kg/m^2^ in the fracture group and 22.9 kg/m^2^ in the non-fracture group (*p* = 0.118). There was no significant difference in the proportion of patients with a BMI less than 18.5 kg/m^2^ (underweight) and those with a BMI above 30 kg/m^2^ (severe obesity) (*p* = 0.623, *p* = 1.000). There were no significant differences in the medication history for osteoporosis, drinking, smoking, diabetes mellitus, rheumatoid arthritis, hypertension, or dialysis between the two groups (Table 1).

### 3.2. Basic Characteristics According to the Presence or Absence of BMD Discordance

The basic characteristics of the three groups were compared: the group without BMD discordance (no discordance), the group with discordance in which BMD of the hip was lower than that of the spine (low hip group), and the group with discordance in which BMD of the spine was lower than that of the hip (low spine group). The mean age was 77.6 years in the no discordance group, 78.1 years in the low hip group, and 73.5 years in the low spine group (*p* = 0.030). There were no significant differences in the height or sex of each group. The average weight was 55.6 kg in the no discordance group, 56.4 kg in the low hip group, and 62.8 kg in the low spine group (*p* = 0.006), but there were no differences in BMI (*p* = 0.135). There were no differences in the history of medication for osteoporosis, smoking, rheumatoid arthritis, diabetes mellitus, and hypertension in each group. Regarding drinking history, there was a significant difference between the groups. A total of 5.4% of the patients in the no discordance group, 10.3% in the low hip group, and 28.6% in the low spine group were alcohol drinkers (*p* = 0.003) (Table 2).

### 3.3. Analysis of the Results of Osteoporosis Tests

The average femoral T-scores were −2.61 and −2.26 for the fracture group and non-fracture group, respectively, indicating a significant difference (*p* = 0.015). The T-score of the lumbar spine was −1.87 in the fracture group and −1.99 in the non-fracture group (*p* = 0.512). The quantified value of the femoral bone density was 0.540 g/cm^2^ and 0.570 g/cm^2^ (*p* = 0.084) and the bone density of the spine was 0.794 g/cm^2^ and 0.764 g/cm^2^ in the fracture and non-fracture groups, respectively (*p* = 0.347). T-score discordance occurred in 58 patients (53.2%) in the fracture group and 31 patients (28.4%) in the non-fracture group (*p* < 0.001). The difference between the T-score of spine and hip was 1.08 in the fracture group and 0.69 in the non-fracture group (*p* < 0.001).

The type of discordance was divided into no discordance, minor discordance, and major discordance. The fracture group had a ratio of 51:50:8 patients (46.8%, 45.9%, and 7.3%), and the non-fracture group had a ratio of 78:31:0 patients (71.6%, 28.4%, and 0%), respectively, indicating that the fracture group had a significantly higher discordance rate (*p* < 0.001). The type of discordance was divided into cases with no discordance, low hip discordance, and low spine discordance. In the fracture group, there was a ratio of 51:47:11 patients (46.8%, 43.1%, and 10.1%), and in the non-fracture group, there was a ratio of 78:21:10 patients (71.6%, 19.3%, and 9.2%), respectively (Table 3).

The odds ratio (OR) for hip fracture was 2.861 times higher in patients with discordance than in the no discordance group (95% CI: 1.633–5.015; *p* < 0.001). The odds ratio (OR) of hip fracture was 3.423 times higher in the low hip discordance group than in the no discordance group (95% CI: 1.834–6.388; *p* < 0.001). The odds ratio (OR) of hip fracture was higher in the low spine discordance group than in the no discordance group, but the difference was not significant (OR = 1.682; 95% CI: 0.666–4.248; *p* = 0.268) (Table 4).

## 4. Discussion

Hip fracture represents a significant health concern among the elderly population, with high associated mortality rates and functional disabilities. Screening, interpretation, and treatment of osteoporosis are becoming a major issue, with primary care physicians paying greater attention to the increase in osteoporosis patients [11]. However, since BMD discordance is relatively managed for patients without fractures, studies regarding treatment of osteoporotic fracture patients, especially hip fracture patients, are lacking [12]. In addition, it is not yet clear whether there is a significant difference in T-score discordance between patients with hip fracture and patients without hip fracture. Moreover, there have been only a limited number of comparative studies that have investigated differences in T-score discordance between these two groups.

Since Alireza et al. introduced the discordance concept of osteoporosis in 2005, many studies have been conducted [10]. In 2008, Howard et al. mentioned that the interpretation of osteoporosis should be approached in a site-specific manner [13]. Since then, various studies have presented BMD discordance in the country from which they have been reported [14]. BMD discordances are known to occur in up to 50% of cases [14]. When Seok et al. introduced a study on BMD discordances in South Korean patients, they showed a lot of interest in South Korea [15], and Hong et al. reported that it occurs in 12.9% of men over 50 years of age and 10% of postmenopausal women [2]. In this study, 53.2% of hip fracture patients and 32.1% of patients without hip fractures had BMD discordance, which is significantly higher than in other studies. Since we analyzed patients with a severe underlying disease who required hospitalization, it is likely that more BMD discordance was noted in them compared to outpatients. Alarkawi et al. reported a 30% higher risk of vertebral fracture in women over 60 years of age when there was a discordance in which the vertebral BMD was lower than that of the hip, compared to the case in which the femoral BMD was lower (HR: 1.30, 95% CI: 1.11 to 1.45) [5]. That study showed the significance of BMD discordance.

According to a previous article, five distinct factors contributing to T-score discordance have been documented [16]. Physiological discordance arises from the natural adaptive responses of the skeletal system to both external and internal factors and forces. This form of discordance manifests as variations in total hip BMD between the dominant and non-dominant hips [17]. Another instance of physiological discordance arises from differential rates of bone loss at various skeletal sites as individuals age [18]. The peak bone mass of the spine is attained over five years prior to that of the hip, exerting a significant influence on T-score discordance between these two regions. Pathophysiological discordance emerges as a consequence of underlying medical conditions, with common examples including vertebral osteophytosis, vertebral end-plate and facet sclerosis, osteochondrosis, and aortic calcification [19,20]. Anatomical discordance is linked to variations in the composition of the bone envelope, as evidenced by T-score differences between the posteroanterior lumbar spine and the supine lateral lumbar spine within the same patient. Artifactual discordance may occur when dense synthetic objects, like zippers or clips, are present within the field of view during the test. Lastly, technical discordance may arise when the technician improperly positions the patient for the test or when there are hardware or software issues affecting the acquisition of test data. While the specific reasons for the elevated prevalence of T-score discordance in hip fracture patients could not be definitively explained based on these five factors, it is hypothesized that the combined effects of physiological and pathophysiological discordance played a role. This assumption stems from the advanced age of our patient cohort (average age: 77.9 years), surpassing that of the sample population in previous reports [15,21].

Currently, clinicians do not meaningfully interpret and manage BMD discordance. In other words, the treatment is based only on the BMD of the lowest region. In addition, there are no guidelines on how to manage BMD discordance and explain its concept to patients. Currently, there are various types of drugs for osteoporosis, and they are administered in various ways depending on the patient’s sex, age, and fracture site. Particularly, in the case of hip fractures, bone formation promoters should be administered rather than bone resorption inhibitors, and even some of the same bisphosphonates may not be suitable for treatment. In this study, BMD discordance was investigated in patients with hip fractures, and the prevalence and type of discordance were analyzed. This study is meaningful in that it reveals the significance of BMD discordance in hip fracture patients compared to patients without hip fracture. The difference in BMD between the hip and spine was greater, and there were more patients with low hip discordance among the hip fracture patients than among the patients without hip fracture. Therefore, low hip discordance among the BMD discordance types is a risk factor for hip fracture.

The treatment of osteoporosis should be multifaceted, and treatment is currently being conducted in various clinical departments. However, due to the characteristics of each department, the interpretation of BMD is different, and thus the prevention and treatment methods are different. The purpose of this study was to present an index that can be used by primary care physicians who are not easily exposed to cases of hip fracture for the interpretation and treatment of BMD discordance between the hip and spine of hip fracture patients and for providing guidance for the prevention of future hip fractures in orthopedic joint patients.

There are some limitations to this study. First, our study is constrained by a relatively small sample size, and the non-random or non-representative selection of patients. Consequently, caution must be exercised when attempting to generalize the prevalence of BMD discordance among hip fracture patients based on our findings. The limited sample size may not fully capture the broader population’s diversity of characteristics and risk factors. Second, it would be difficult to represent all patients due to the diversity of diseases at the time of admission in patients without hip fractures. While we implemented a 1:1 propensity match to control for age, gender, and BMI when selecting the control group, it is important to acknowledge that not all potential confounding factors influencing fractures were accounted for in our study [2]. Third, the history of medication for osteoporosis is a factor that can significantly influence the interpretation of BMD, and the two groups only included a few patients with a history of medication use (fracture group: 12 patients [11.0%]; non-fracture group: 10 patients [9.2%]). Only medical records were referenced, and there were cases for which the investigation of medication at the time of hospitalization may not be adequate; therefore, it may be difficult to interpret BMD if there are more patients with a history of medication. Fourth, given that this study adopted a cross-sectional design, it is important to note that it did not capture changes in BMD occurring before and after the occurrence of fractures. This limitation raises the potential for reverse causality, where the observed differences in BMD could, in some cases, result from the fracture itself rather than serving as a causal factor. Future prospective studies will be able to overcome this limitation.

## 5. Conclusions

Patients with hip fractures had a lower mean BMD than those without hip fractures. There were more cases of BMD discordance between the hip and spine in patients with hip fracture than in patients without hip fracture. The difference in BMD between the hip and spine was greater and there were more patients with low hip discordance among the hip fracture patients than among the patients without hip fracture. Therefore, low hip discordance among the BMD discordance types is a risk factor for hip fracture. In the presence of low hip discordance, more active treatment and preventive measures for hip fractures are necessary.

## Figures and Tables

**Table 1:** The general characteristics of the study population.

	Fracture Group (*n* = 109)	Non-Fracture Group (*n* = 109)	*p* Value
Age (mean ± SD)	77.9 ± 6.9	76.9 ± 7.5	0.284
Sex (Male:Female, %)	36:73 (33.0%:67.0%)	38:71 (34.9%:65.1%)	0.775
Height (cm, mean ± SD)	158.5 ± 8.2	157.8 ± 9.0	0.499
Body weight (kg, mean ± SD)	55.9 ± 9.5	57.2 ± 9.8	0.334
BMI (kg/m^2^, mean ± SD)	22.3 ± 3.2	22.9 ± 3.1	0.118
BMI < 18.5 (*n*, %)	10 (9.2%)	8 (7.3%)	0.623
BMI > 30 (*n*, %)	3 (2.8%)	3 (2.8%)	1
Medication for osteoporosis (*n*, %)	12 (11.0%)	12 (11%)	1
alcohol (*n*, %)	10 (9.2%)	10 (9.2%)	1
smoking (*n*, %)	11 (10.0%)	8 (7.3%)	0.471
DM (*n*, %)	32 (29.4%)	23 (21.1%)	0.16
RA (*n*, %)	1 (0.9%)	4 (3.7%)	0.175
Hypertension (*n*, %)	67 (61.5%)	55 (50.5%)	0.102
Dialysis (*n*, %)	2 (1.8%)	1 (0.9%)	0.561

BMI, body mass index; DM, diabetes mellitus; RA, rheumatoid arthritis; fracture group: patients hospitalized for hip fracture; non-fracture group: patients hospitalized without hip fracture.

**Table 2:** The general characteristics of the discordance groups.

	No Discordance	Discordance		
	No Discordance (*n* = 129)	Low FN Discordance (*n* = 68)	Low LS Discordance (*n* = 21)	*p* Value
Age (mean ± SD)	77.6 ± 7.4	78.1 ± 6.5	73.5 ± 7.1	0.03
Sex (M:F, %)	40:89	24:44	10:11	0.316
	(31.0%:69.0%)	(35.3%:64.7%)	(47.6%:52.4%)	
Height (cm, mean ± SD)	157.4 ± 8.6	158.5 ± 8.3	162.0 ± 9.4	0.069
Body weight (mean ± SD)	55.6 ± 9.1	56.4 ± 9.6	62.8 ± 11.1	0.006
BMI (kg/m^2^, mean ± SD)	22.4 ± 3.0	22.5 ± 3.4	23.8 ± 3.1	0.135
BMI < 18.5 (*n*, %)	10 (7.8%)	7 (10.3%)	1 (4.8%)	0.686
BMI > 30 (*n*, %)	2 (1.6%)	3 (4.4%)	1 (4.8%)	0.425
Medication for osteoporosis (*n*, %)	14 (10.9%)	9 (13.2%)	1 (4.8%)	0.553
Alcohol (*n*, %)	7 (5.4%)	7 (10.3%)	6 (28.6%)	0.003
Smoking (*n*, %)	10 (7.8%)	7 (10.3%)	2 (9.5%)	0.827
DM (*n*, %)	30 (23.3%)	21 (30.9%)	4 (19.0%)	0.398
RA (*n*, %)	4 (3.1%)	1 (1.5%)	0 (0%)	0.585
Hypertension (*n*, %)	76 (58.9%)	36 (52.9%)	10 (47.6%)	0.522
Hemodialysis (*n*, %)	0 (0%)	3 (4.4%)	0 (0%)	0.035

FN, femoral neck; LS, lumbar spine; BMI, body mass index; DM, diabetes mellitus; RA, rheumatoid arthritis.

**Table 3:** The statistical differences between two groups according to the results of DEXA.

	Fracture Group (*n* = 109)	Non-Fracture Group (*n* = 109)	*p* Value
T-score of proximal femur	−2.61 ± 0.91	−2.26 ± 1.18	0.015
T-score of lumbar spine	−1.87 ± 1.87	−1.99 ± 1.86	0.512
BMD of the femoral neck (g/cm^2^)	0.54 ± 0.11	0.57 ± 0.14	0.084
BMD of the lumbar spine (g/cm^2^)	0.794 ± 0.12	0.764 ± 0.15	0.347
Discordance (*n*, %)	58 (53.2%)	31 (28.4%)	<0.001
Discordance (T-score)	1.08 ± 0.88	0.69 ± 0.64	<0.001
Classification of discordance (no:minor:major, %)	51:50:8 (46.8%:45.9%:7.3%)	78:31:0 (71.6%:28.4%:0.0%)	<0.001
Type of discordance (no:low FN:low LS, %)	51:47:11 (46.8%:43.1%:10.1%)	78:21:10 (71.6%:19.3%:9.2%)	<0.001

BMD, bone mineral density; FN, femoral neck; LS, lumbar spine; fracture group: patients hospitalized for hip fracture; non-fracture group: patients hospitalized without hip fracture.

**Table 4:** Odds ratio (OR) for hip fracture of each discordance group, compared with no discordance group.

Characteristics	OR	95% CI		*p*-Value
Discordance	2.861	1.633	5.015	<0.001
Low FN discordance	3.423	1.834	6.388	<0.001
Low LS discordance	1.682	0.666	4.248	0.268

FN, femoral neck; LS, lumbar spine.

## Data Availability

The datasets used and/or analyzed during the current study available from the corresponding author on reasonable request.
